# Supplementary material for: Real-World Treatment Efficacy and Safety Profile of Sofosbuvir- and Velpatasvir-Based HCV Treatment in South Korea: Multicenter Prospective Study
Source: Viruses. 2025 Jul 4;17(7):949. doi: 10.3390/v17070949 (PMC12300531; doi:10.3390/v17070949)
Supplement: Supplementary file 1 [file viruses-17-00949-s001.zip › viruses-3672036-supplementary.pdf]

Supplementary Table S1. Longitudinal changes of liver fibrosis score in patients who were treated with sofosbuvir plus velpatasvir (n=99).

	APRI		FIB-4 index		LSM	
	Mean (95% CI)	*p*-value	Mean (95% CI)	*p*-value	Mean (95% CI)	*p*-value
Initial	1.09 (0.95–1.23)	reference	4.28 (3.64–4.92)	reference	10.68 (8.73–12.64)	reference
4w	0.48 (0.33–0.63)	<0.001	2.96 (2.28–3.63)	<0.001		
8w	0.43 (0.25–0.62)	<0.001	2.83 (1.98–3.68)	<0.001		
ETR	0.49 (0.33–0.64)	<0.001	2.92 (2.19–3.64)	<0.001		
SVR12	0.48 (0.29–0.63)	<0.001	2.86 (2.10–3.62)	<0.001	9.66 (6.40–12.92)	<0.001

APRI, AST to platelet ratio; FIB-4, fibrosis-4; LSM, liver stiffness measurement; ETR, end of treatment response; SVR12, sustained virological response at 12 weeks

Supplementary Table S2. Longitudinal changes of liver fibrosis score in patients who were treated with sofosbuvir, velpatasvir plus voxilaprevir (n=16).

	APRI		FIB-4 index		LSM	
	Mean (95% CI)	*p*-value	Mean (95% CI)	*p*-value	Mean (95% CI)	*p*-value
Initial	1.51 (1.06–1.97)	reference	4.72 (3.33–6.12)	reference	10.50 (5.39–15.61)	reference
4w	0.67 (0.19–1.15)	0.001	3.40 (1.90–4.89)	0.004		
8w	0.64 (0.07–1.21)	0.001	3.55 (1.78–5.32)	0.005		
ETR	0.51 (0.03–0.99)	<0.001	2.66 (1.17–4.15)	0.004		
SVR12	0.50 (0.31–0.68)	<0.001	2.50 (1.82–3.19)	<0.001	6.81 (0.70–12.92)	0.048

APRI, AST to platelet ratio; FIB-4, fibrosis-4; LSM, liver stiffness measurement; ETR, end of treatment response; SVR12, sustained virological response at 12 weeks

Supplementary Table S3. Clinical data about patients with decompensated liver cirrhosis and treatment outcome

Patient Number	Child-Pugh Score at baseline	Event of decompensation	Treatment outcome	Child-Pugh score after treatment
1	10	Esophageal varix<br>Ascites (Grade 3)	Improvement of ascites	5
2	8	Ascites (Grade 2)	Improvement of ascites	5
3	10	Ascites (Grade 2)<br>Hyperbilirubinemia (Total bilirubin 7.77mg/dL)	Improvement of ascites and hyperbilirubinemia	5
4	7	Ascites (Grade 1)<br>Hyperbilirubinemia (Total bilirubin 3.25 mg/dL)	Remaining ascites (Grade 1) and hyperbilirubinemia (2.09mg/dL)<br>Recurrence of multinodular HCC	6
5	7	Ascites (Grade 1)	Improvement of ascites	5

*All patients reached sustained virological response at 12 week

Supplementary Table S4 . Clinical data about patients with prior history of hepatocellular carcinoma and outcome after anti-viral therapy

Patient Number	Interval from last HCC treatment to start of anti-viral treatment (months)	Treatment modality for HCC	HCC outcome after anti-viral treatment
1	6	TACE	No recurrence after SVR (19 months)
2	1	TACE	Recurrence at 6th week of treatment initiation
3	8	RFA	No recurrence after SVR (12 months)
4	8	DEB-TACE	No recurrence after SVR (8 months)
5	10	RFA	No recurrence after SVR (18 months)
6	4	TACE	Recurrence after SVR (3 month)
7	8	TACE, RFA, Proton therapy	Recurrence at ETR

1~6th patients were treated with sofosbuvir plus velpatasvir and 7th patient was treated with sofosbuvir, velpatasvir, plus voxilaprevir. All patients achieved SVR.

HCC, hepatocellular carcinoma; TACE, trans-arterial chemoembolization; RFA, radio-frequency ablation; DEB, drug-eluting bead; ETR, end of treatment response; SVR, sustained virological response

Supplementary Figure S1. Flowchart of the study.

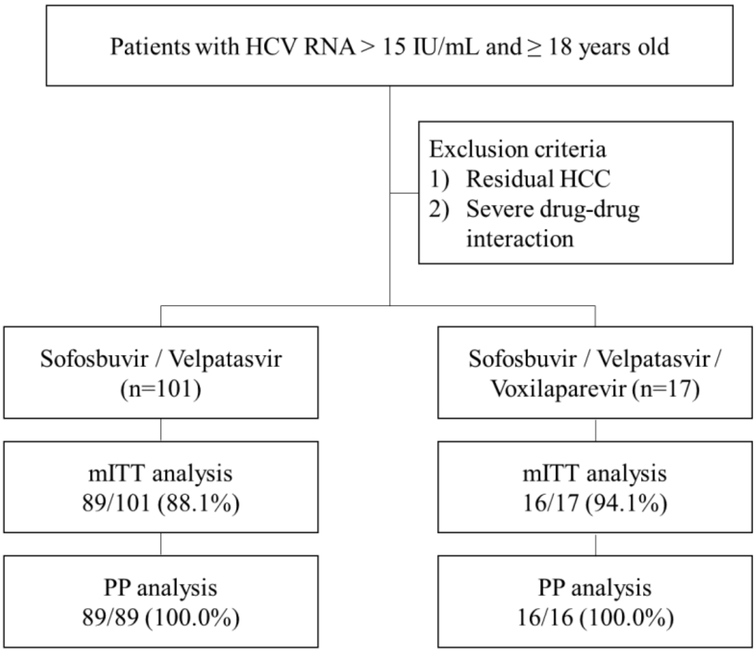

HCV, hepatitis C virus; HCC, hepatocellular carcinoma; mITT, modified intention-to-treat; PP, per protocol
